# Editorial: A Matter of Bottom-Up or Top-Down Processes: The Role of Attention in Multisensory Integration

**DOI:** 10.3389/fnint.2017.00005

**Published:** 2017-02-28

**Authors:** Jess Hartcher-O'Brien, Salvador Soto-Faraco, Ruth Adam

**Affiliations:** ^1^Perceptual Intelligence Lab, Industrial Design Engineering, Delft University of TechnologyDelft, Netherlands; ^2^Institució Catalana de Recerca i Estudis AvançatsBarcelona, Spain; ^3^Departament de Tecnologies de la Informació i les Comunicacions, Universitat Pompeu FabraBarcelona, Spain; ^4^Institute for Stroke and Dementia Research, Klinikum der Universität München, Ludwig-Maximilians-Universität (LMU)Munich, Germany

**Keywords:** attention, bottom-up, congruency, cross-modal, multisensory integration, top-down

Our everyday environments are multisensory and our brains handle this rich information in an extremely efficient way. Yet, attention's role in the process of multisensory integration (MSI) is still the object of intense debate. Whilst some evidence supports that attention guides MSI via top-down selection of inputs, others suggest that bottom-up integration can occur pre-attentively capitalizing on temporal and spatial correlations. Understanding the role of attention in MSI is further complicated by the fact that attention itself refers to a variety of different selection mechanisms. Thus, the interplay between attention and MSI can take many forms and lead, as evident in the literature, to mixed findings and apparent contradictions (e.g., Driver, 1996; Talsma et al., 2010; Jack et al., 2013; Macaluso et al., 2016). This *Frontiers in Integrative Neuroscience* Research Topic aims at helping clarify the nature of this interplay by posing a specific and narrow question. The reader will find a collection of 10 empirical papers plus an opinion and a review article which can broadly be classified into those addressing the contribution of bottom-up processing in MSI, and those exploring top-down modulations.

One framework for exploring the interplay between attention and MSI is to assume that attention leads to reweighting of sensory information (Bresciani and Ernst, 2007). Focusing on *bottom-up* contributions, Vercillo and Gori address such potential reweighting using the Maximum Likelihood Estimate model. The effect of attention on the weighting of sensory information could be disentangled by measuring observer's audio-tactile spatial estimates, showing that bottom-up attention increases precision and alters sensory weighting in MSI. These findings corroborate selective attention's role in adjusting the brain's computations for achieving an integrated multisensory percept. Keeping the bottom-up perspective, Hazan et al. address visual search in the barn owl. Similar to ventriloquism (e.g., Pick et al., 1969) and visual search in humans (Onat et al., 2007), the owls' visual search behavior was modulated by sound, demonstrating that audio-visual interactions guided visual attention. Visual search mechanisms might be similar among mammalian and non-mammalian species, owing to correlations between visual and auditory events in nature. An ultimate demonstration of the effect of bottom-up processes would consist in showing MSI for sub-threshold stimuli, in the absence of top-down biases. Aller et al. take a step in this direction showing that the visibility of a visual event under continuous flash suppression (CFS) increases when a sound is congruent (instead of incongruent). Albeit, as the authors argue, possible top-down processes may still exert an influence, the CFS framework provides a clear conceptualization of the question of bottom-up versus top-down processes. Jones' study explores both attentional cuing via bottom-up temporal entrainment and spatial cuing of attention in unisensory and cross-modal events. Both temporal and spatial attention-MSI interactions facilitated behavioral responses: attention produced a response advantage when deployed in a bottom-up temporal-cuing fashion and via top-down spatial attention manipulations. However, there was no measurable interaction between the bottom-up and top-down processes observed.

This research topic also includes two review/opinion papers with different views on bottom-up MSI (De Meo et al.; Talsma). De Meo et al. interpret expressions of early multisensory interaction as *integration*. Such that integration phenomena are irreducible, albeit top-down control processes can regulate their expression. Talsma et al. instead argue that cross-modal interactions that take place early, requiring no role of attention, do not result in *integration*. This controversy suggests that we may be missing crucial evidence, or are looking at extant evidence from incongruous angles. Defining what is meant by “integration” would already be an important step in the right direction.

Despite the attempts to find core, bottom-up MSI interactions, top-down attentional components may also determine the outcome of MSI (e.g., Aller et al.). Whether these influences are general, or confined to specific contexts, is still a matter of debate. This Research Topic includes five articles that have identified *top-down* influences employing various manipulations of multisensory *congruency*.

In an attempt to disentangle bottom-up versus top-down contributions Donohue et al. manipulate attentional load and observer goals. Audio-visual binding in the bounce-stream paradigm was modulated by spatial cueing, suggesting that attention alters temporal binding of audio-visual signals in this task. Attention produced a response advantage when deployed in a bottom-up temporal-cuing approach and via the top-down spatial attention manipulation. However, similar to Jones' conclusion (Jones), there was no measurable interaction between bottom-up and top-down processes. Employing an audio-visual congruency manipulation with the attentional blink paradigm, Adam and Noppeney could show that task-irrelevant sounds influence detection of, and awareness to, a visual target. Increased awareness of visual inputs was based not only on the congruency of current sensory evidence but also on prior knowledge, hinting that top-down expectations affect decisions regarding multisensory events and enhance integration. Mastroberardino et al. addresses whether task-irrelevant stimuli modulate cross-modal processing of semantically-congruent cues, by neutralizing low-level contributions. Consistent with the idea of extensive processing of cross-modal semantic relations, their fMRI results reveal that semantic-congruency engages fronto-parietal networks related to visuo-spatial control. Consequently, one could think of semantic congruency as providing a bias signal that exerts influence (yet not dominance) on the competitive interplay between bottom-up and top-down processes for the control of processing resources. Once one accepts that top-down influences are pervasive in MSI, the question of content-dependency arises. Su's study explores to what extent content congruency will determine low levels of information processing in MSI and illustrates that audio-visual correspondence relations derived from human movements exert an important influence on auditory deviant detection and even on cross-modal synchrony perception.

The relation between attention and MSI further increases in complexity when manipulating stimulus-elicited *emotions*. Only a few studies have investigated multisensory emotion processing, despite the importance of both emotions and MSI to adaptive behavior. Takagi et al. establish that attentional instructions and audio-visual congruency modulate sensory dominance in emotion processing. This study highlights how important it is to provide participants with detailed and clear instructions when characterizing MSI-attention interactions. Finally, Doose-Grünefeld et al. find no direct relationship between MSI and attention. Their study also investigates MSI of emotional signals, yet in patients with depression. The patients rated faces as more fearful when displayed with happy sounds and appeared impaired in processing positive auditory information even when task-irrelevant. Neurocognitive tests revealed that those patients had impaired attention, which was not related to their emotion perception. Thus, impaired attention cannot directly explain deficits in multisensory (emotional) processing.

## Conclusion and where do we go from here

The work presented in this Research Topic demonstrates that the relation between MSI and attention is complex and unlikely to be answered by one single study. By bringing together these diverse works we observe that stimulus context effects, such as spatial/temporal co-location (e.g., Hazan et al.) or semantic (e.g., Mastroberardino et al.) and emotional congruency (e.g., Takagi et al.), as well as the goal of the observer, such as changing task for similar stimuli (e.g., Donohue et al.; Jones) tend to characterize whether MSI will be modulated by top-down attentional effects (e.g., Adam and Noppeney; Mastroberardino et al.; Talsma) or will seem to occur preattentively (e.g., Aller et al.; De Meo et al.; Hazan et al.; Su; Vercillo and Gori). It is fair to say that the interplay depends on many factors and, in some situations, involves no direct relation between attention and MSI (e.g., Doose-Grünefeld et al.). Clearer and universally agreed definitions would limit the same results being used for different perspectives on the debate of attention's role in MSI. Future research using standardized instructions and experimental designs, e.g. CFS, controlling for either bottom-up or top-down influences (or both) across different contexts and observer goals would help get closer to a resolution of this ongoing debate.

## Author contributions

JH, SS, and RA co-editored the Research Topic and wrote the editorial.

### Conflict of interest statement

The authors declare that the research was conducted in the absence of any commercial or financial relationships that could be construed as a potential conflict of interest.

## References

[B1] Bresciani J. P. Ernst M. O. (2007). Signal reliability modulates auditory–tactile integration for event counting. Neuroreport 18, 1157–1161. 10.1097/WNR.0b013e3281ace0ca 17589318

[B2] Driver J. (1996). Enhancement of selective listening by illusory mislocation of speech sounds due to lip-reading. Nature 381, 66–68. 10.1038/381066a0 8609989

[B3] Jack B. N. O'Shea R. P. Cottrell D. Ritter W. (2013). Does the ventriloquist illusion assist selective listening? J. Exp. Psychol. Hum. Percept. Perform. 39, 1496–1502. 10.1037/a0033594 24079471

[B4] Macaluso E. Noppeney U. Talsma D. Vercillo T. Hartcher-O'Brien J. Adam R. (2016). The curious incident of attention in multisensory integration: bottom-up vs. top-down. Multisens. Res. 29, 557–583. 10.1163/22134808-00002528

[B5] Onat S. Libertus K. König P. (2007). Integrating audiovisual information for the control of overt attention. J. Vis. 7:11. 10.1167/7.10.11 17997680

[B6] Pick H. L. Jr. Warren D. H. Hay J. C. (1969). Sensory conflict in judgments of spatial direction. Percept. Psychophys. 6, 203–205. 10.3758/BF03207017

[B7] Talsma D. Senkowski D. Soto-Faraco S. Woldorff M. G. (2010). The multifaceted interplay between attention and multisensory integration. Trends Cogn. Sci. 14, 400–410. 10.1016/j.tics.2010.06.008 20675182 PMC3306770

